# Coding Dyadic Behavior in Caregiver–Child Interaction from a Clinical Psychology Perspective: How Should Multiple Instruments and Outcomes Be Dealt with?

**DOI:** 10.3390/children10111765

**Published:** 2023-10-31

**Authors:** Anne Jung, Nina Heinrichs

**Affiliations:** Department of Psychology, Clinical Child and Adolescent Psychology and Psychotherapy, Bielefeld University, P.O. Box 100131, 33501 Bielefeld, Germany; nina.heinrichs@uni-bielefeld.de

**Keywords:** caregiver–child interaction, dyadic interaction, behavioral observation, child development

## Abstract

The experiences children have in the interactions with their caregivers influence their developmental outcomes. To target caregiving and optimize intervention effects, the assessment of caregiver–child interactions is highly relevant for families affected by parental mental disorders. Behavioral observation is a widely used method for assessing family dynamics, and the literature offers a wide variety of instruments with which to code such data. However, a structured overview of behavioral observation instruments (BOIs) is lacking, and the multitude of types of dyadic behaviors (DBs) assessed within each BOI are complicating their application. We aim to provide an overview of the BOIs applied to families affected by mental disorders and suggest a DB taxonomy that may be used across BOIs. We first conducted a systemic literature search to identify the most frequently used BOIs and the DBs they capture in clinical psychology. Second, we asked 13 experts to sort DB terms based on perceived conceptual similarity and analyzed these results using multidimensional scaling. We found approximately 450 different terms for DBs, and we argue that DBs can be classified within two overarching dimensions, i.e., in terms of structure and in terms of reaction to a child’s signals. These efforts can facilitate the coding and application of BOIs in clinical practice.

## 1. Introduction

The quality of caregiving that children receive is crucial for their further development. Their experiences in interactions with their caregivers have a tremendous impact on a multitude of psychosocial outcomes [1]. For instance, they lay the foundation for attachment quality [2] and further socio-emotional development (such as externalizing and internalizing problems) [3], cognitive development [4], and children’s self-concept and self-esteem [5].

Considering the relevance of caregiver–child interactions, identifying families’ problems on an interactional level is essential in order to support caregivers in building up and maintaining a positive relationship with their child. For the identification of both strengths and difficulties in the interaction between caregiver and child, behavioral observation instruments (BOIs) are considered a gold standard by many researchers [6]. Depending on the perspective taken (e.g., such as clinical psychology, educational, or developmental psychology) and the goal associated with observing these interactions, the resulting BOIs may be different. These instruments generally allow for a simultaneous assessment of behavior on different levels, namely, on the level of the caregiver, the level of the child, and the level of the dyad [7]. Such a detailed assessment of the dyadic interaction provides information about problems and resources for both individuals and can therefore be used in a resource-oriented treatment approach in clinical psychology. At the same time, it enables the identification of difficulties in the interaction and can also give an impression of the symptomatology of both individuals [8]; hence, it is also useful for treatment planning [9]. 

### 1.1. Challenges in Selecting a Suitable BOI

However, selecting an appropriate BOI for one’s own research or clinical question is a difficult task. One of the most prominent challenges is the multitude of existing BOIs to choose from [10]. The selection process is marked by the opaque nature of the BOI landscape, which previous studies have already attempted to address, each with a specific question for narrowing down fitting BOIs. For instance, Aspland and Gardner [6] reviewed BOIs that are useful for observing interactions involving children with conduct problems, while Lotzin et al. [7] focused on psychometric studies. Wiefel et al. [8] gave an overview of BOIs, which can be used to characterize behavioral problems among children aged 0 to 5 years; Horowitz et al. [11] concentrated on early mother-child interactions. Other researchers only included BOIs measuring sensitivity [12], BOIs available in specific languages [10,13,14], or instruments for the assessment of dyadic behavior during mealtime [15]. However, none of the aforementioned studies provided a comprehensive overview of BOIs in general or guidelines on how to accumulate knowledge from the various different BOIs, e.g., for conducting a meta-analysis.

### 1.2. Challenges in Selecting Dyadic-Behavior-Related Terms of Interest

Apart from the necessity of choosing one BOI from among many, selecting a fitting instrument for one’s own clinical or research question requires extensive knowledge about each construct assessed with the respective BOI [7]: information about the construct itself and (if applicable) its embedment in a broader theoretical model, its interconnectivity with other constructs assessed, and its relevance for one’s own primary outcome all need to be processed and evaluated. Acquiring such comprehensive knowledge is almost impossible considering the large number of different terms used in BOIs to describe dyadic behavior. For example, Müller et al. [10] identified up to 320 different terms, even when focusing on BOIs available exclusively in the German language. These terms are also notable for their lack of linguistic consistency (e.g., “sensitivity”, “sensitive responsiveness”, and “responsivity”), with a failure to reference the terms’ theoretical foundations [10]. We are not aware of any comprehensive research investigating the operational definitions of similar terms, how they are similar or distinctive in relation to each other, and which associations they have with developmental outcomes. While previous research has addressed the lack of consistent use of terms as well as the lack of clarity about the connections between similar terms, these issues have been addressed with a specific focus. De Wolff and van IJzendoorn [2], for example, asked experts to categorize different terms related to sensitivity and a child’s attachment, while other researchers focused on disentangling terms related to synchrony [16] or explored the associations between dyadic behavior terms assessed with three different BOIs [17]. Jacob [13] listed several aspects of dyadic behavior that emerged from an overview of BOIs available in German. When looking more closely at the sorting procedures executed by these researchers, some similarities emerge: in several cases, mutuality, synchrony, a positive attitude, affectionate parenting, and emotional support or supportive parenting are named as relevant terms. At the same time, however, many discrepancies persist, and the terms are identified in only one sorting procedure without establishing a counterpart (e.g., stimulation [2], self-enjoyment parenting or overwhelmed parenting [17], mirroring, attunement, or reparation [16]). A proposal for a taxonomy including a variety of BOIs embedded in different paradigms, such as attachment theory or social learning theory, has not yet been made. Rather, research has concentrated on specific clusters of the caregiver–child interaction such as sensitivity or synchrony.

The identification of suitable structures for describing dyadic behavior can be carried out via different (statistical) methods. Cluster and factor analyses are probably the most likely to be used in this context (e.g., as applied by Unternaehrer et al. [17]). Other approaches include asking experts to sort terms based on their perceived similarity [2] or systematically analyzing terms using text analysis software [16]. Each approach for identifying a meaningful structure in dyadic behavior or for simplifying the relationship between dyadic behaviors is associated with different terms of the output (e.g., “cluster”, “factor”, or “dimension”, to name a few). A “factor” describes dyadic behaviors that are highly correlated with each other and thus classified into a group that is distinct from other identified factors [18]. In contrast, a cluster analysis aims to classify variables in “clusters” in which the dyadic behavior terms within a cluster are as similar as possible but, at the same time, as different as possible from the terms in another cluster [18]. Both “factors” and “clusters” therefore describe a group of dyadic behaviors associated with each other; however, this closeness is identified by applying different statistical methods. A third option, related to cluster analyses, is the multidimensional scaling (MDS) approach [19], which can be used when a concept of underlying dimensions is lacking [20]. MDS groups dyadic behaviors based on the spatial distance between terms in the n-dimensional space [21]. Here, the output is named a “dimension”, and its meaningfulness is determined by the dyadic behaviors’ coordinate values [21]. Thus, a “dimension” describes a construct that distinguishes between different dyadic behaviors. A “dimension” is more overarching in nature and can be used to understand the structures underlying different “factors” or “clusters”, while these describe shared characteristics in a group of related dyadic behaviors.

While different methods serve as the basis for structuring dyadic behavior and developing a taxonomy, terms are also used to describe behaviors at different levels of abstraction [7], ranging from specific and direct behavior (e.g., gazing and laughing) to more global aspects and latent constructs (e.g., sensitivity and intrusiveness). Some authors also differentiate between (meta-)theoretical concepts (e.g., mutuality) and specific processes (e.g., contingency) [16], opening further levels of abstraction. Researchers have not yet investigated whether and how these different levels of abstraction are intercorrelated. Moreover, it remains unclear if the level of assessment leads to different associations with child development.

### 1.3. Aim of the Present Study

Currently, each step taken in the process of selecting a fitting BOI reveals the lack of (1) a comprehensive overview of suitable BOIs and which dyadic behaviors have been investigated by which researchers and (2) a taxonomy for the classification of terms used in these BOIs. One primary aim of the present study was, therefore, to identify which BOIs are most frequently used in clinical contexts. The other primary aim was to develop a suggestion for a taxonomy on how to classify dyadic behavior terms. We not only aimed to identify clusters of dyadic behavior (i.e., groups of related dyadic behaviors) but also dimensions (i.e., overarching information on how to structure dyadic behaviors and distinguish between different clusters).

## 2. Materials and Methods

We used two different, independent approaches to achieve our goals: First, as part of another study, we conducted a systematic review and meta-analysis using the main research questions (not included in this report), targeting dyadic behavior among mothers with mental disorders. (The original research questions we planned to address with the meta-analysis (see PROSPERO CRD42022314240 [22]) are not the subject of this report. We are currently in the process of preparing the main outcome related to the a priori developed research questions for publication [23].) During this meta-analytical process, we also obtained a great amount of information on the types of BOIs used and the frequency with which they were employed and assessed dyadic behaviors, and we took advantage of the database to extract this information in the clinical context. Based on this information, herein, we provide an overview of the instruments and terms included in this research (excluding child behavior codes). Since the systematic search conducted for the meta-analysis focused on the dyadic interactions of mothers suffering from mental disorders, it should be noted that the search strategy was limited to this field of research and excluded, for example, BOIs used in non-clinical settings as well as research (and its associated BOIs) focusing on father–child interactions. 

Second, we already anticipated an excessive number of terms describing dyadic behavior based on the literature. We also aimed to draw upon expert knowledge to develop guidelines for classifying dyadic behavior. Therefore, using a second method, we asked experts to group terms from the BOIs identified in the existing literature based on their perceived similarity. This list of BOIs was generated independently from the database based on the meta-analytic search, as described above. We will now describe these two strategies in more detail. 

### 2.1. Systemicatic Identification of BOIs and Dyadic Behaviors Using a Meta-Analytic Search

Although we will not describe the primary outcomes of the meta-analysis here, a brief outline of our search and selection strategies is nonetheless relevant for contextualization since the generated database for calculating frequencies of BOIs and dyadic behaviors is dependent upon inclusion criteria. For another study with different research questions [22], we conducted a systematic review and meta-analysis to investigate the dyadic behavior of caregivers with different mental disorders. To avoid adding to the heterogeneity of studies in the meta-analysis while also allowing for the inclusion of a wide range of disorders, we focused exclusively on mothers rather than fathers in the systematic search. This procedure was selected because the majority of studies (still) examine mothers rather than fathers and mothers are often a child’s primary caregiver. For the identification of BOIs, this procedure has little relevance because BOIs are usually not developed to target one specific gender. Nonetheless, the search focused exclusively on mothers suffering from mental disorders. We used the resulting literature as a basis for identifying BOIs. After consultation with a librarian, three databases (MEDLINE, PsycINFO, and PubMed), with two additional citation-based databases (Scopus and Web of Science) were searched for relevant articles in October 2021. The search terms are displayed in Table S1. If applicable, the databases’ thesauri were used to specify the search parameters. Before conducting the final search, we met with a librarian to pre-test and validate the search.

For the primary research question to be addressed in the meta-analysis, we included only empirical studies written in English or German, with at least two groups of mothers who were observed while interacting with their child. At least one group had to consist of mothers who had been diagnosed with a mental disorder during their child’s life (clinical group), while the comparison group could include either a group of mothers with a different mental disorder (clinical control group) or a group of mothers without a clinical diagnosis (control group). This focus on studies with a control group design targeting mothers with mental disorder(s) was chosen as part of the meta-analytical research question (not included in this report). Studies were excluded if (a) the behavioral observation only occurred after an intervention or prior to group assignment (i.e., before the assessment of mental disorders), (b) no direct behavioral information was assessed in the dyadic situation (e.g., spatial proximity), or (c) a preselected sample was used (e.g., prenatal selection for substance abuse and later assessment of depression). We also excluded studies (d) with fewer than 10 individuals per group or (e) studies whose data structures did not allow for the extraction of the targeted information for the main research questions of the meta-analysis (e.g., if the results were grouped according to the child’s age). 

Two independent researchers (the first author and a research assistant) first double-screened each study and, afterwards, extracted relevant data using the Covidence software product [24]. Emerging discrepancies were resolved in consultation with a third person (last author) in a consensus process after evaluating each rating together. We identified roughly 6700 studies fitting the search parameters and screened 3668 articles for titles and abstracts. Out of these 3668 articles, 1063 articles were assessed for eligibility, of which we included a total of 278 studies. Of these 278 studies, 170 studies were suitable for examining dyadic behavior as a categorical outcome and BOIs in more detail; the descriptive results regarding BOIs presented in the Results section thus refer to *n* = 170 studies. 

Regarding dyadic behavior, we extracted the numbers and terms of assessed dyadic behaviors along with the type of situation in which the behavioral observation took place and its length; for behavioral observations in multiple situations, this information was extracted per situation. Additionally, we extracted the BOIs’ names and the types of observation (micro- vs. macro-observation). After extraction, we prepared and analyzed the data using R [25].

### 2.2. Similarity Sorting Task

Independently from the meta-analysis, we adapted the Similarity Sorting Task (SST) approach developed by De Wolff and van IJzendoorn [2]. For the SST, experts in the field of caregiver–child interaction were asked to sort pre-selected terms based on their perceptions of their similarity and group them in conceptually meaningful ways. Experts included researchers as well as clinicians from clinical and developmental psychology who were instructed to select as few groups with high conceptual similarity as possible. Since our goal was to reduce complexity, we specified a maximum of 10 possible groups in which the terms could be sorted and asked the experts to name their sorted groups. The experts were informed that the groups did not necessarily have to include the same number of terms.

We pre-selected 38 terms for sorting and used the names of scales from 6 frequently used BOIs in the literature with different underlying paradigms: the Child Adult Relationship Experimental Index (CARE, *n* = 3 scales) as cited in [26], the Coding Interactive Behavior System (CIB, *n* = 3 scales) as cited in [27], the Dyadic Parent–Child Interaction Coding System (DPICS, *n* = 16 scales) [28], the Emotional Availability Scales (EAS, *n* = 4 scales) [29], Ainsworth’s Maternal Sensitivity Scales (MSS, *n* = 4 scales) [30], and the BOI used in the National Institute of Child Health and Human Development study (NICHD, *n* = 8 scales) [31]. Most BOIs are grounded in attachment theory (CARE, EAS, MSS, and NICHD), while the CIB also has some roots in attachment theory but focuses on a broader spectrum of dyadic behavior, and the DPICS is based on social learning theory. The DPICS is also the only instrument that uses a micro-coding approach, which also explains the increased number of terms identified, as micro-coding instruments typically assess smaller defined aspects of dyadic behavior.

As recommended by Wood and Wood [32], we provided additional information on each term using the respective scale descriptions published in the literature or taken directly from the manuals (if available). In some cases (CARE and CIB), if we could not find uniform descriptions, we selected the articles that contained the most information. In contrast to De Wolff and van IJzendoorn [2], we did not use cards but a PowerPoint template for the terms and an additional PDF document with the additional descriptions.

A convenience sample of 21 experts was recruited via e-mail by both authors. All in all, 13 German experts completed the SST, each of them working either in research or in practice with caregivers and their children at the time. Three experts were developmental psychologists, while the other ten worked in clinical psychology. A sample size of 10 to 15 sorters was assumed to be sufficient for further multidimensional analysis [33,34]. 

The experts’ classification was transferred to SPSS [35] and analyzed using an MDS approach, which is recommended when analyzing card-sorting data [21,34]. MDS belongs to the family of multivariate techniques and transforms mathematical relationships between stimuli, i.e., in our case, between the sorted terms, into spatial relationships. For this purpose, the frequency of co-occurrence of the terms in the same piles, across the different experts’ classifications, was first converted into a 38 × 38 matrix, and the distance between the individual stimuli was estimated via the MDS approach. The resulting proximity matrix consisted of Euclidean distance measures reflecting dissimilarity between the stimuli [21]. As stimulus coordinates, these Euclidean distances provide information about the spatial distance between different stimuli in the n-dimensional space [21]. By plotting the coordinates, this spatial distance or proximity can be visually represented, with stimuli perceived as more similar being located close to one another and stimuli perceived as less similar being located further apart [19,33]. By calculating goodness-of-fit parameters, the optimal number of n dimensions can be identified. Following the recommendations of Whaley and Longoria [21], we calculated R^2^ as a goodness-of-fit measure and Kruskal’s stress index as a badness-of-fit parameter to identify the optimal number of dimensions for the sorted terms. The optimal number was selected by evaluating changes in fit measures from n dimensions to n-1 dimensions and assessing the interpretability of the dimensions. R^2^ is an indicator of the amount of variance explained, with higher values representing greater variance explained [21], meaning that higher values are preferred. In contrast, in Kruskal’s stress index a lower value indicates a better fit [20,21]. A stress index less than or equal to 0.10, or a negligible change when transitioning from n to n-1 dimensions, are considered indicators of a good fit. 

In accordance with previous research [21], we further calculated a quotient measure of both fit indices representing the change between dimensions. As an example, for R^2^, for dimension change 1, we divided the R^2^ of dimension 1 by the R^2^ of dimension 2 and applied the same procedure for the stress indices and the four other dimension changes, respectively. A quotient of 1 indicates that there is no difference between the n and n + 1 dimension solutions [21]. MDS was performed in SPSS using the Alternating Least Squares Approach to Scaling (ASCAL), and figures were created using R [25]. A detailed description of how to perform MDS has been provided by Whaley and Longoria [21]. 

## 3. Results

### 3.1. Systemicatic Identification of BOIs and Dyadic Behaviors

The systematic identification of the most frequently used BOIs in clinical psychology also provided us with information about the terms used within these instruments. We therefore not only give an overview of the BOIs but also of the dyadic behaviors assessed.

#### 3.1.1. Overview of BOIs 

Out of the 170 studies, we identified *N* = 121 distinctive BOIs in total. Since in some cases more than one BOI was used per study, a total of 208 coding instruments were used in the 170 studies. All instruments that were applied more than once are shown in Table 1, while an overview of all the BOIs—applied once or more—is provided in Table A1. 

Table 1 demonstrates that CARE, CIB, and PCERA were the most frequently used BOIs for assessing maternal dyadic behavior when at least one group of mothers with a mental disorder was included in the primary studies. The ages of their children varied from 12 days to 18 years, although the majority of the studies focused on younger children (*mean* = 3.421 years; *SD* = 0.621 years). Overall, however, when comparing Table 1 and Table A1, it is noticeable that the majority of BOIs were only applied once. Of the total 121 instruments identified, only 24 were used more than once in the primary studies, as displayed in Table 1. In most publications (*n* = 97), either new instruments or adaptations of two or more instruments were used, which we listed as a new category. Additionally, of the 97 instruments used once, it became apparent that many authors (*n* = 18 in total, see Table A1) developed new instruments rather than relying on existing instruments.

#### 3.1.2. Overview of Dyadic Behavior Terms

**Figure 1:**
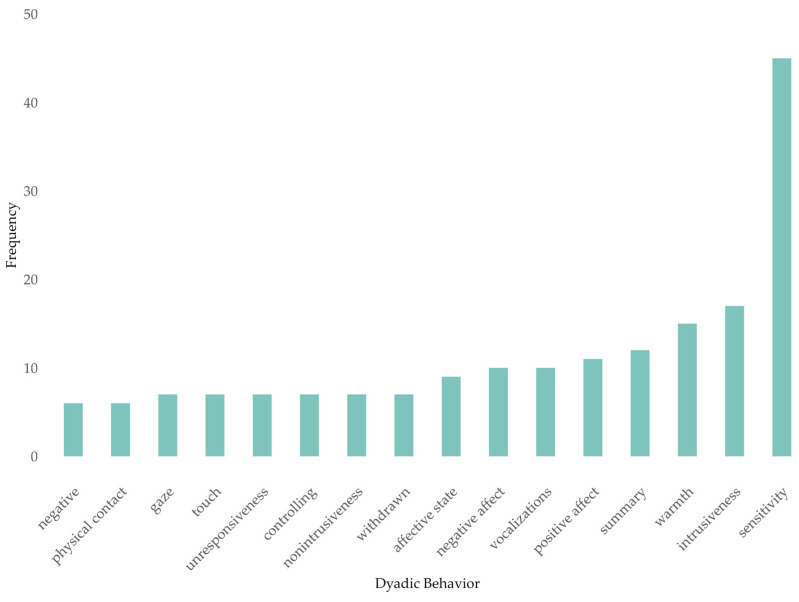
Most frequently used dyadic behaviors.

As anticipated, a large number of different terms were identified for dyadic behavior. Based on the entire set of 170 studies, roughly 450 distinctive terms were extracted. As displayed in Figure 1, sensitivity was by far the most frequently assessed dyadic behavior, and it was included in 6.164% of the primary studies’ outcomes. Intrusiveness (2.329%) and warmth (2.055%) were also assessed quite frequently; the construct intrusiveness was assessed even more frequently than the figure suggests at first sight since the opposing pole non-intrusiveness was registered separately as a term in the primary studies and, accordingly, is also listed separately here. Terms describing dyadic behavior on different levels of abstraction are apparent. Both macro- and micro-analytic terms are represented among the most frequently assessed dyadic behaviors, with macro-analytic terms (e.g., sensitivity, intrusiveness, etc.) seeming to be more common than micro-analytic ones (e.g., vocalizations, touch, gaze, etc.). 

Comparable to the results regarding BOIs, it was also noticeable in the case of dyadic behaviors that the majority of terms were examined only once in the 170 primary studies (*n* = 353 terms assessed once vs. *n* = 89 terms assessed multiple times). Across all the studies considered, a total of 730 dyadic behaviors were recorded; accordingly, the majority of studies examined more than one dyadic behavior.

### 3.2. Dyadic Behaviors and Dimensions from the Similarity-Sorting Task

The values of the SST’s fit parameters are presented in Table 2.

**Figure 2:**
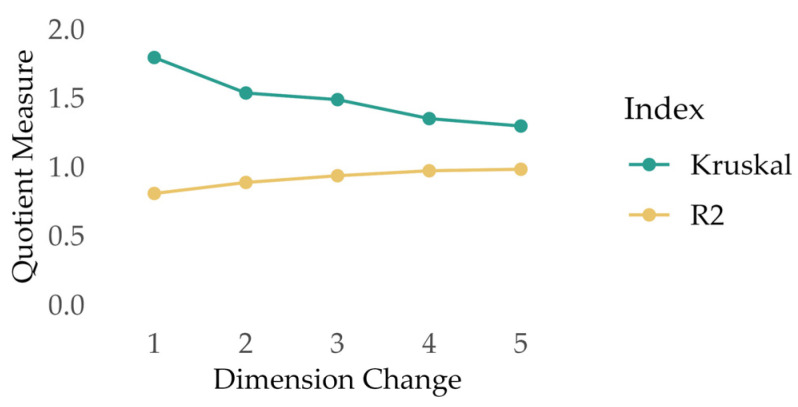
Quotient measures of SST’s fit parameters for dimension changes. Note. A quotient of 1 indicates no difference between the n and n + 1 dimension solution.

The quotient measures of the fit parameters are plotted in Figure 2 and demonstrate, on the one hand, that two- or three-dimensional solutions both indicate a good fit and, on the other hand, that adding more dimensions does not lead to a sufficient improvement in the fit parameters. Accordingly, a two- or three-dimensional solution is preferable. 

**Figure 3:**
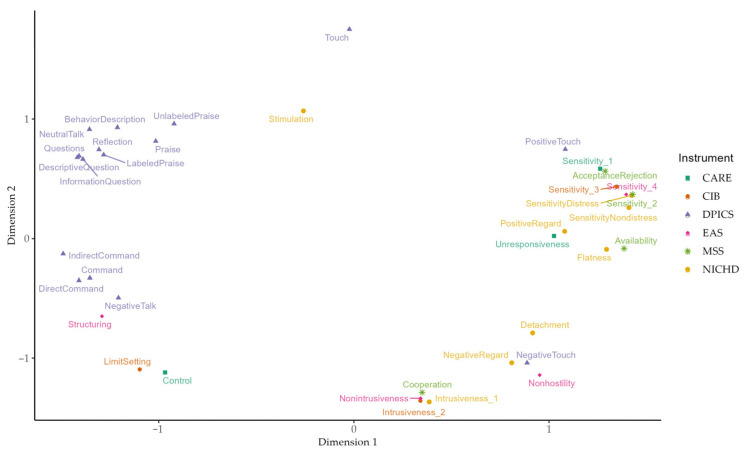
Original SST solution with two dimensions.

Since we aimed to reduce complexity, we primarily present the two-dimensional solution here. However, the three-dimensional solution can be viewed in the Supplementary Material (Figure S1). The two-dimensional solution is displayed in Figure 3.

For a better understanding of the dimensions’ meanings, we examined the stimulus coordinate values (see Table S2). Other researchers recommended focusing on absolute values greater than 1.5 to capture the dimensions’ meaning [34]. Since most of our absolute values were less than 1.5, we considered terms with absolute values greater than 1.4 as more meaningful for the dimensions’ interpretation, with negative values indicating the meaning of one pole and positive values indicating the meaning of the other pole, respectively.

The highest values of **Dimension 1** are *IndirectCommand*, *DescriptiveQuestion*, *Direct Command*, and *Questions* on one pole and *Sensitivity_2*, *SensitivityDistress*, and *SensitivityNondistress* on the other pole. We classified these two poles as “structured” and “non-structured” respectively, with “structured” representing more active, directive, and guiding dyadic behavior and “non-structured” reflecting more permissive behavior that follows a child’s signals. We named this first dimension “structure”. 

One pole of **Dimension 2**, on the other hand, is best described by *Intrusiveness_1*, *Intrusiveness_2*, *Nonintrusiveness*, *Cooperation*, *Nonhostiliy*, and *Control*. This pole represents dyadic behavior characterized by a restriction of a child’s behavior (“child restriction”). It seems striking at first that *Intrusiveness* and *Nonintrusiveness* were loaded onto the pole with the same loading directions. However, when considering the experts’ task, this no longer seems unusual. In most cases, descriptions of the same behavior, whether formulated positively or negatively, were assigned to the same pile, from which, in turn, stimulus coordinates were estimated within the MDS.

The second pole of Dimension 2 is loaded primarily with *Touch* and to a lesser degree with *Stimulation*, *UnlabeledPraise*, *BehaviorDescription*, and *Praise*. This pole was labeled “child reinforcement and encouragement”. It represents the reinforcing behavior of a caregiver, through which child behavior is described, extended, or reinforced. The dyadic behavior characterized by this pole is more reactive than active in nature and is a response to a child’s signals. In consideration of these two poles, dimension 2 was called “reaction to child’s signals”. 

**Figure 4:**
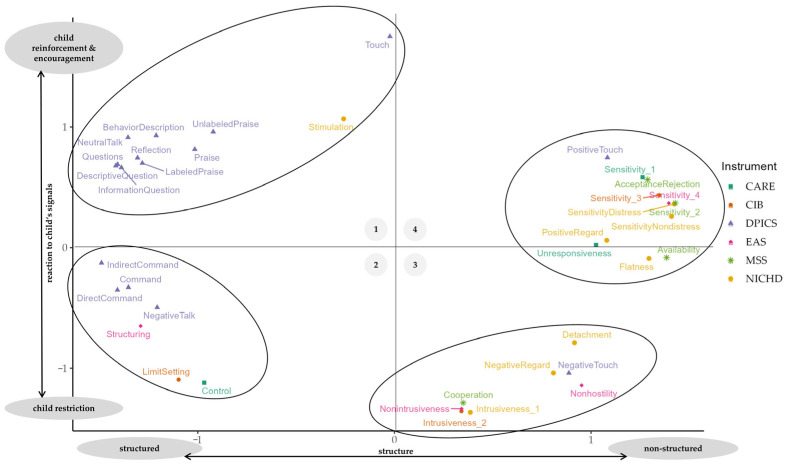
SST solution with the two dimensions “structure” and “reaction to child’s signals”.

After applying these dimension labels to Figure 3 and taking a closer look at the terms displayed, four separate groups emerged from the data. For the groups found in our SST, we use the term “cluster”, even though we did not perform a cluster analysis in a strict sense. However, since there are some parallels between the analysis methods and as the comprehensibility has increased, we have chosen this term. The dimensions’ labels, including their quadrants with the four clusters to be observed, are shown in Figure 4.

The quadrant in the upper left (cluster 1) contains terms that characterize more structured behavior (e.g., *BehavioralDescription*, *Stimulation*, etc.) as well as encouraging, supportive behavior (e.g., *Praise*, *Reflection*, etc.). It therefore describes structured and encouraging dyadic behavior. It mainly contains dyadic behavior terms from the DPICS; NICHD’s *Stimulation* is the only item sorted into this cluster from a different BOI. The other clusters contain terms from different BOIs. The quadrant in the lower left (cluster 2), exhibits restrictive dyadic behavior that is simultaneously characterized by a high degree of structure, e.g., in the form of boundary setting and controlling behavior. In turn, the quadrant in the bottom right (cluster 3) also displays behaviors that can be described as restrictive, such as intrusiveness and hostility. However, these behaviors are less structuring and more reactive in nature than their counterparts in cluster 2. Detached behavior can additionally be found in cluster 3; overall, we labeled cluster 3 as representing restrictive yet non-structured dyadic behavior. Finally, the top right quadrant (cluster 4) describes responsive rather than self-induced behavior, in which children’s signals are followed and, at the same time, encouraged and supported. Accordingly, it contains terms such as sensitivity and responsiveness. Hence, this quadrant represents encouraging, non-structured dyadic behavior.

## 4. Discussion

This study aimed to provide an overview of commonly used instruments for coding parental dyadic behavior and to investigate which kind of dyadic behavior is assessed most frequently in clinical psychology. Finally, due to a lack of a common taxonomy for terms describing dyadic behavior, we additionally suggested a taxonomy for structuring these terms. To accomplish these research aims, we first employed a systematic literature search that was conducted as part of a meta-analysis [22]. Overall, out of the 170 included studies, we identified 121 distinctive BOIs, which roughly assessed 450 dyadic behaviors in clinical psychology. Most instruments were used only once, and the majority of dyadic behaviors were also investigated in only one study. However, more commonly used BOIs, such as Crittenden’s CARE [60], Feldman’s CIB [37], Clark’s PCERA [61] and Murray’s GRS [39], could be identified. Across these instruments, sensitivity, intrusiveness, and warmth were the most frequently examined dyadic behaviors. Second, we performed a sorting task, asking experts to sort terms for dyadic behavior based on perceived similarity, and analyzed these data using a multidimensional scaling approach. Two dimensions were identified as being helpful for structuring the opaque dyadic behavior landscape. One dimension describes the structure and covers the range from structured (i.e., more active and directive caregiving) to less-structured behavior, the latter of which is characterized by being more permissive in nature. The second dimension represents the extent to which caregivers align their behavior with children’s signals. It therefore describes, at one end, rather restrictive behavior that is barely oriented toward children’s signals, and at the other end, supportive behavior that encourages a child. With the help of these two dimensions and their poles, behaviors could be clustered into (1) structured and encouraging dyadic behavior, (2) structured and restrictive dyadic behavior, (3) non-structured and restrictive dyadic behavior, and (4) non-structured and encouraging dyadic behavior.

### 4.1. Systemicatic Identification of BOIs and Dyadic Behaviors 

Despite the meticulous preparation of the meta-analytical search, several limitations of our study need to be taken into consideration. On the one hand, the original research question of the meta-analysis influenced the identification of BOIs and dyadic behaviors. Since the meta-analysis was intended to provide more detailed information on dyadic behavior among mothers with different mental disorders, we systematically searched for studies including mothers as well as studies with at least one control group (either with or without a different mental disorder compared to the study group) [22]. Studies focusing on behavioral observation regarding fathers or other caregivers as well as studies without control groups or without at least one study group with a mental disorder were therefore excluded. The data presented above on BOIs and dyadic behaviors thus only draw on a specific subset of the existent literature that specifically focuses on mothers with mental health issues. Accordingly, the frequency results are limited in their generalizability, which is reflected in the fact that BOIs frequently used in other contexts, such as education, only appear with a low frequency of use in the present study (e.g., FOS and HOME; cp. Table A1). For a broader systematic identification of BOIs and dyadic behaviors, an adapted search strategy might have been more suitable, e.g., one where the search was not limited to mothers and mental disorders specifically and where databases used in educational or developmental sciences were searched additionally. However, in a clinical context, a systematic search provides valuable information nonetheless and can result in useful guidelines for (clinical) researchers and practitioners alike, helping them to obtain a better understanding of the instruments that exist and the kinds of behavior typically assessed across a broad range of mental health issues affecting caregivers.

On the other hand, we included several terms for dyadic behavior (e.g., sensitivity, warmth, etc.) as well as BOIs (e.g., CIB, EAS, PCERA, CARE, etc.) in our search strategy found in an unstructured a priori literature search. The predefinition of such specific instruments or behaviors may have had an influence on frequency in the outcome such that precisely these instruments and dyadic behaviors were also found more frequently in the literature. Other behaviors identified less frequently in the primary studies, such as “stimulation” or “praise”, might have appeared more frequently in the final literature subset if specific search terms for these dyadic behaviors had been included in the search terms. However, the search terms were intentionally added to find more appropriate studies and were based on the aforementioned unsystematic literature search conducted in advance. Accordingly, it is reasonable to assume that the BOIs and dyadic behaviors listed in the search criteria would have appeared frequently even without their incorporation into the search strategy. Moreover, the specified criteria did not need to appear in a study’s title, abstract, or keywords for it to be identified as being potentially suitable for the research question.

Furthermore, when extracting the BOIs, we noticed that some BOIs were named unclearly. Especially in earlier publications but also for self-developed instruments, it was often the case that no common names for the instruments were given. These instruments were typically listed only in reference to the authors’ names, and these same instruments were often referred to with different names. Given that, in some cases, these authors published lots of research on caregiver–child interaction and also frequently used behavioral observation, it was not always clear whether the instruments were the same or new. We therefore were forced to cross-check the references in each study by hand. Since the coding manuals of the respective instruments were not published in an open access manner, we were not able to cross-check every instrument referred to in a similar way. If an insufficient amount of detail about a BOI’s scale was described, the instrument was listed as new. In some cases, therefore, it cannot be ruled out that the instrument initially labeled in the primary studies according to the author and year was later given a common name and thus listed more than once. This might lead to slight distortion in the frequencies with which the BOIs were presented. However, since many instruments were only used with a low base rate, the effect is probably rather small. In addition to such difficulties concerning the correct identification of identical BOIs, some primary studies referred to a commonly used instrument but noted that adaptations had been made for the purposes of the study. In many cases, more than one BOI was adapted in parallel for the assessment. These BOIs could also only be listed separately, so some instruments might be underrepresented in Table 1.

Finally, the interpretation of the results should consider that the quality of the included studies has not yet been checked and that possible publication biases have not been reported so far. This information is currently in preparation and will be published as part of the meta-analysis [23].

### 4.2. Similarity Sorting Task

The dimensions identified in the SST did not emerge in other attempts at structuring dyadic behavior terms [2,13,16,17], although some commonalities can be found with respect to the clusters. It is not surprising that our dimensions are not reflected well in previous research because most research to date has studied clusters rather than dimensions. Even though De Wolff and van IJzendoorn [2] identified two dimensions, they tended to look at the clusters’ content and did not describe the dimensions further. The dimensions, nonetheless, are similar to the core principles of behavioral parenting programs based on the *Hanf Model* [62,63]. They usually distinguish between child- and parent-directed interactions, with child-directed interactions reflecting dimension 2, which clusters caregiver’s behaviors in response to their children’s signals. In contrast, during parent-directed interactions, caregivers are usually required to be active, e.g., by providing clear rules using appropriate commands. This principle is reflected in the first dimension, which clusters caregivers’ behaviors based on the degree to which they are structured [62,63]. Similarly, Baumrind classified caregivers’ behavior into high responsiveness (vs. low warmth) and high demandingness (vs. low control or strictness) and developed categories of parenting styles through different combinations of these dimensions [64]. Baumrind’s first dimension, high responsiveness, is quite similar to the pole “child reinforcement and encouragement” of our second dimension since it describes behavior implying that a caregiver is involved in their child’s activities and supports them [64]. Baumrind’s demandingness represents the amount of parents’ controlling behavior, for example, in the form of implementing rules or setting standards for behavior [64,65], and therefore reflects aspects of our first dimension, “structure”, as well as the second pole of dimension 2, namely, “child restriction”. 

To the best of our knowledge, our proposed taxonomy is the first to identify dimensions using a multidimensional analysis. Investigating underlying dimensions, however, provides additional information on how to structure dyadic behavior. Besides grouping dyadic behaviors by content (e.g., via a text analysis program [16]) or correlations (e.g., via a factor analysis [17]), an exploration of underlying dimensions offers further insight into constructs that differentiate or resemble these clusters in terms of content. Since no other study, to the best of our knowledge, has identified underlying dimensions, it was not possible for us to draw on existing labels. The naming of the dimensions identified in our study thus unintentionally contributes to the multitude of different labels. In order to avoid increasing this multiplicity even further, we took care to maintain the same naming structure when naming the clusters.

The identified clusters in the present study are in line with previous research and share several commonalities. The first cluster (“structured and encouraging dyadic behavior”), for instance, is both similar to a cluster called “stimulation” [2], which includes behavior focusing on the encouragement and stimulation of a child, and a factor named “supportive parenting” (including affirmative parenting, paying attention to a child, affective involvement, and verbalization) [17]. Yet, our cluster also contains dyadic behavior, which was sorted in the study by De Wolff and van IJzendoorn [2] into a different cluster, namely, “emotional support”. Here, the experts classified dyadic behavior such as a supportive presence or behavior, stimulation, and assistance [2]. Thus, the experts in De Wolff and van IJzendoorn’s study [2] made more of a distinction between the affective and stimulating component of encouragement compared to the experts in our sorting task. Despite the fact that our SST and subsequent analysis are similar to their counterparts in De Wolff and van IJzendoorn [2], a direct comparison between the identified clusters is not straightforward because the experts were asked to sort dyadic behavior described in different terms. Hence, the differences in the resulting clusters could also be due to the different inputs. For example, the biggest difference with respect to these terms is that our sorting task did not include terms such as involvement or playfulness. The differences in sorting were likely due to the different inputs available in the SST. The advantage of our input is its higher variation of the terms’ underlying concepts. While De Wolff and van IJzendoorn [2] referred to terms describing parental behavior in relation to Ainsworth’s sensitivity concept [66] as well as parental behavior in relation to child attachment, we included terms beyond this concept, e.g., labeled praise, touch, or negative regard for a child. In contrast, the input used by De Wolff and van IJzendoorn [2] has the advantage of more finely differentiated constructs that were included for sorting. Accordingly, a more accurate picture of the content shared between clustered terms is possible. In addition, terms for synchrony and mutuality were included, which were missing in our work (see below).

When comparing our work to that conducted by Unternaehrer et al. [17], it can be noticed that the factor “supportive parenting” further entails dyadic behavior such as insensitivity and intrusiveness or negative affect, which were classified differently in our study. Instead of being sorted into the cluster “structured and encouraging dyadic behavior”, these terms were perceived as more similar to other behaviors indicating “non-structured and restrictive dyadic behavior” (our third cluster). Since the SST of De Wolff and van IJzendoorn [2] only used terms closely related to Ainsworth’s concept of sensitivity [66], no comparable term was identified. In terms of content, our cluster is reflected in the social learning paradigm, especially in the *Early Childhood Coercion Model*, which focuses on harsh parenting [67], and in attachment theory, where intrusiveness is addressed as the opposite of sensitivity.

Our fourth cluster (“non-structured and encouraging dyadic behavior”) is comparable to “positive attitude” [2] since both terms cluster dyadic behavior describing acceptance, positive affect and affectivity, attention, and sensitivity together. These assortments differ from all the factors delineated by Unternaehrer et al. [17], where the aforementioned dyadic behaviors were sorted into different factors. Hence, in Unternaehrer et al.’s work [17], sensitivity can be found under “supportive parenting” as well as “affectionate parenting”. Looking at the BOIs’ terms’ loadings’ in this cluster, instruments grounded in attachment theory were the most commonly identified. In terms of content, the cluster also describes primarily dyadic behavior, which can be attributed to the core construct of attachment theory, that is, sensitivity [66].

One cluster that seems to be lacking in our study compared to previous research is a cluster that entails dyadic behaviors focusing on synchrony [2,16]. Since none of the included terms for our SST described dyadic behavior in terms of synchrony or mutuality, our experts were not able to sort dyadic behaviors based on this information. This is likely attributable to a lack of using BOIs specifically related to synchrony, as they did not end up in the most frequently used list of the BOIs. Also lacking were terms describing a caregiver’s relationship satisfaction with their child or their mental health; these terms were, however, included in the form of questionnaire data in a factor analysis [17], and they represent more extended constructs besides direct dyadic behavior.

In sum, the differences between the clusters probably result from the different inputs: while De Wolff and van IJzendoorn [2] did not provide information on how exactly the terms were selected for the SST and Unternaehrer et al. [17] used a mix of questionnaire and behavioral observation data for a factor analysis, we used terms employed in widely used BOIs. In addition, Unternaehrer et al. [17] also conducted a factor analysis on collected parent–child dyadic data, whereas, in our case, the experts were explicitly asked to sort terms based on their conceptual similarity. Thus, the (statistical) conditions under which the clusters emerged are substantially different, which may also explain the differences in the results. The additional information on how to sort dyadic behaviors is valuable and deepens our knowledge of behavioral observation and dyadic behavior, e.g., with respect to connections between different terms of dyadic behavior and a broader understanding of relevant clusters or constructs that need to be considered.

There are several limitations to consider when interpreting the SST’s results. The commencement of the systematic search for the meta-analysis and the conception of the SST occurred simultaneously. Since data extraction of the search results took nearly two years, we were not able to use the information from the systematic identification for the SST’s conception. Therefore, we did not include the terms of some BOIs that we identified to be among the most frequently used instruments, such as PCERA, GRS, and IRS. Also, the BOIs included in the SST were mostly based on attachment theory. DPICS is the only instrument with roots in social learning theory, and it is also the only BOI using a micro-analytical assessment. Most of the input given to the experts to sort was therefore macro-analytical and based on attachment-related terms. Considering the identified clusters, it seems striking that the first cluster (“structured and encouraging dyadic behavior”) solely consists—with one exception, namely, the term *Stimulation* from the NICHD scales— of DPICS terms. One possible explanation for this could be that the experts had difficulty integrating the different levels of abstraction, and therefore many DPICS terms were sorted together based on the level of abstraction rather than on the similarity of their content. In contrast, some DPICS terms were also sorted into other clusters (e.g., positive touch was sorted into cluster 4, “non-structured and encouraging dyadic behavior”). The only other BOI for which terms were mainly sorted into one cluster was the MSS. This loaded mostly on cluster 4, “non-structured and encouraging dyadic behavior”. A closer look at the scope of this instrument explains this sorting quite easily, as the MSS mainly captures maternal sensitivity. Cluster 4 is where all the terms with sensitivity in their name were sorted by the experts, so it seems unsurprising that the MSS terms are mainly found in this cluster (the exception is *Cooperation*, which was sorted into cluster 3, “non-structured and restrictive dyadic behavior”). The other clusters consisted of terms from several BOIs and CARE, CIB, EAS, and NICHD loaded in different clusters.

As mentioned above, the original manuals of the BOIs were frequently unavailable; this was also the case for some of the instruments and terms used in the sorting task. In accordance with the study by De Wolff and van IJzendoorn [2], we provided additional information on the terms using descriptions of the scales from the manuals. However, since we were not able to attain information on each term, we occasionally had to use information from other publications quoting the manual. These additional descriptions, however, did not always provide valuable information. Providing the respective manuals’ original descriptions of the terms might have been helpful in the sorting task. In addition, access to the manuals would have been helpful to identify all the terms of an instrument. In the case of the CIB, for example, different terms were mentioned in different publications, which is why we only selected those that were listed at least twice for the input for the SST.

Finally, the composition of the expert sample should also be considered. We made sure to include both practitioners and researchers. It should be noted, however, that all of the experts came from German-speaking countries, while the sorting cards all displayed English terms. An extension to native English-speaking individuals is needed to ensure there is a match between the language of the sorting cards and the native language of the sorters. Additionally, future research should integrate the ratings of experts from educational science. It is possible that native-language-speaking experts as well as experts with different professional backgrounds would sort the dyadic behavior terms differently.

Finally, we received training in the selected BOIs, and we are also trained (or in training) in cognitive-behavioral psychotherapy. We therefore also might have introduced bias in this perspective when processing and interpreting the results. Hence, future research on BOIs may benefit from a more diverse group of researchers.

### 4.3. Practical Implications

Using this overview of BOIs and the proposed dyadic behavior taxonomy might be helpful for the identification of suitable instruments and, subsequently, sorting and structuring the myriad of terms employed, providing useful support for researchers and practitioners alike. Researchers, for example, might find the taxonomy helpful for comparing dyadic data assessed with different instruments, bridging the divide that emerges due to the broad range of different constructs and specific BOIs selected [68]. Our taxonomy provides a first glimpse of how different terms share similar characteristics (in terms of structure or reaction to a child’s signals) and enables an initial assessment of whether they are even comparable using one of the identified clusters. Taking this information as a guideline for sorting different dyadic behaviors in a comparable way could also simplify the performance of meta-analyses or systematic reviews on dyadic data since it narrows down the number of terms used.

Practitioners might especially profit from the overview on BOIs used and the terms assessed in terms of gathering knowledge on which instruments provide helpful information about their clients’ dyadic behavior. While behavioral observations of the interaction between a caregiver and a child in clinical psychology are usually made in the field of child psychotherapy and used for therapy planning [9], interaction with a child is observed much less frequently in adult therapy and related treatment planning. Apart from time restrictions, a lack of knowledge on how to appropriately assess this kind of information might be one reason why behavioral observation is not standard practice in adult psychotherapy despite many adults seeking help being also in primary caregiver roles. Since mental disorders can have an influence on dyadic behavior [69] and as this, in turn, has an influence on a child’s development [70], it may seem reasonable to observe the interaction of adult patients who also have children more frequently. This may help to define therapy goals that include dyadic behavior based on observations, and it also supports interventions enhancing the caregiver–child relationship, which usually has a positive impact on the caregiver’s well-being in return. 

### 4.4. Prospects for Research and Practice

Since our SST offers a lot of potential in terms of shedding more light on a plethora of dyadic behaviors, it would be worthwhile for future research to repeat the task we carried out, paying particular attention to the following issues: (1) The task should be conducted by employing more diverse experts for sorting; ideally, these individuals should not only be German-speaking but should also hail from other countries, and in choosing the experts, both practitioners and researchers should be included. Regarding researchers, it would be preferable to ask both individuals who work with BOIs based on attachment theory and individuals who work primarily with BOIs from social learning theory as well as experts from education and related sciences. (2) In creating the SST, care should be taken to increase the number of BOIs that are based on social learning theory; thus, researchers must endeavor to be more sensitive towards a balance between instruments representing both paradigms. At the same time, more instruments that are commonly used in research—such as PCERA or GRS—should also be included. (3) It would be beneficial to refer to the original manuals in order to better align the descriptions of the terms.

Considering that many manuals are only available after payment and participation in excessive training [7], research and practice would greatly benefit from a freely accessible, comprehensive overview of BOIs, including the dyadic behaviors they cover. Lotzin et al. [7] suggest making manuals collectively available for acquisition from commercial test publishers, similar to the procedure for questionnaire test procedures, so that interested individuals can obtain information about the instrument beforehand. We propose not only collecting this information from commercial test publishers but also from a database, so additional information about the scales and psychometric quality criteria can be added. Moreover, studies using the analyzed instruments could be cross-referenced. Considering the astonishing number of distinctive BOIs identified in the literature [10] as well as in our study, gathering detailed knowledge about each instrument and the dyadic behaviors it captures seems unreasonable. A publicly accessible database would help provide guidance in relation to the multitude of instruments and dyadic behaviors. We have demonstrated that many self-developed instruments are used only once. This could be due to the fact that the developers of these instruments did not know that a suitable BOI already existed and/or thought that it would be necessary to develop a new instrument. A larger database allowing for the possibility of sorting BOIs by the dyadic behaviors they assess could support researchers in such cases and thus counteract the costly development of new and thus rarely used instruments. The information obtained through our systematic search for BOIs and dyadic behaviors can be used as a starting point for the development of such a database. At the same time, it would be useful to conduct the search again with explicit adaptations to behavioral observation in general, without restricting the search to mothers with mental disorders as well as studies with a control group design, as we have described above.

Finally, in order to deepen our knowledge of the interconnectivity between terms for dyadic behavior, it would be interesting to investigate if homonymous terms from different BOIs really do measure the same construct. Therefore, dyadic data should be coded with at least two different BOIs, and the correlation between homonymous terms may need to be analyzed. This, however, is very costly, and it may not be feasible to conduct such a task often (for an example with at least two BOIs assessed, see Unternaehrer et al. [17] or Job et al. [71]). 

## 5. Conclusions

Considering the influence of the quality of a caregiver’s dyadic behavior on the development of their child [70,72], we advocate for a systematic behavioral observation of their interactions in both child and adult psychotherapy. However, particularly for non-experts in the field of behavioral observation, the multitude of instruments and the dyadic behaviors they capture represent a substantial barrier that is hard to overcome. As a guideline on which instrument to code dyadic data with and which dyadic behavior to assess in the first place, we provided an overview of commonly used BOIs and the most frequently assessed dyadic behaviors from a clinical perspective. Nonetheless, it seems reasonable that researchers and practitioners assess different types of dyadic behavior. Sorting and structuring these types of dyadic behavior, therefore, could be helpful for interpreting and categorizing dyadic behavior, such as when one aims to conduct meta-analyses or compare data assessed with different instruments as well as when one is interested in what to look for in clinical practice if the application of a more comprehensive BOI seems impossible. Hence, we propose a taxonomy for structuring caregiver’s dyadic data according to two dimensions, “structure” and “reaction to child’s signals”.

## Figures and Tables

**Table 1:** Most frequently used BOIs.

BOI	BOI (Incl. Author and Publication)	N	Percentage
CARE	Child–Adult Relationship Experimental Index; Crittenden, as cited by Bind et al. [36]	12	5.769
CIB	Coding Interactive Behavior; Feldman [37]	12	5.769
PCERA	Parent–Child Early-Relational Assessment; Clark, as cited by Anke et al. [38]	11	5.288
GRS	Global Rating Scale; Murray et al. [39]	10	4.808
IRS	Interaction Rating Scale; Field [40]	8	3.846
EAS	Emotional Availability Scales; Biringen et al., as cited by Cornish et al. [41]	7	3.365
AFFEX	Automatic Affect Expression System; Izard and Dougherty, as cited by Weinberg et al. [42]	5	2.404
Feeding Scale/SVIA	Feeding Scale; Chatoor et al. [43] (incl. Italian translation, called Scala di Valutazione Interazioni Alimentari/SVIA)	5	2.404
NICHD	Scales from the NICHD study; Owen [31]	5	2.404
MM	Mind–Mindedness; Meins and Fernyhough [44]	4	1.923
MSS	Maternal Sensitivity Scale; Ainsworth [30]	4	1.923
NCAST (NCAF and NCATS)	Nursing Child Assessment Satellite Training; Barnard; as cited by Panzarine et al. [45] Nursing Child Assessment Feeding Scale; Sumner and Spietz, as cited by Minnes et al. [46] Nursing Child Assessment Teaching Scale; Sumner and Spietz, as cited by Page [47]	4	1.923
Appearance Change Codes	Izard, as cited by Reissland and Shepherd [48]	2	0.962
Bakeman and Adamson	[49,50]	2	0.962
BMIS	Bethlem Mother–Infant Interaction Scale; Kumar and Hipwell [51]	2	0.962
Bur	Bur et al., as cited by Righetti-Veltema et al. [52]	2	0.962
GLOS	Greenspan Lieberman Observations System, Greenspan and Lieberman [53]	2	0.962
HOME	Home Observation for Measurement of the Environment; Caldwell and Bradley [54]	2	0.962
ICEP	Infant and Caregiver Engagement Manual; Weinberg and Tronick, as cited by Kaitz [55]	2	0.962
IFIRS	Iowa Family Interaction Rating Scales; Melby et al., as cited by Jaser et al. [56]	2	0.962
MBS	Mannheimer Beurteilungsskala zur Erfassung der Mutter–Kind Interaktion; Jörg et al., as cited by Hohm et al. [57]	2	0.962
Monadic Phases	Monadic Phases; Tronick et al. [58]	2	0.962
POSER	Play Observation Scheme and Emotion Rating; Wolke, as cited by Hipwell et al. [59]	2	0.962

**Table 2:** Fit parameters of the SST.

	Number of Dimensions					
	1	2	3	4	5	6
R^2^	0.606	0.754	0.853	0.914	0.943	0.962
Kruskal’s Stress Index	0.412	0.230	0.150	0.101	0.075	0.058

Note. Interpretation of R^2^: higher values represent greater amount of variance explained. Interpretation of Kruskal’s Stress Index: Index ≤ 0.10 represents a better fit.

## Data Availability

The data and some additional material presented in this study are openly available in an OSF repository at DOI 10.17605/OSF.IO/J2X7R.

## Supplementary Materials

The following supporting information can be downloaded at https://www.mdpi.com/article/10.3390/children10111765/s1. Figure S1: SST solution with three dimensions, Table S1: Search strategy for the identification of relevant studies, and Table S2: Stimulus coordinates of the SST terms.

## Appendix A

**Table A1:** Frequency of BOIs’ usage in primary studies.

BOI	BOI (Incl. Author and Publication)	N	Percentage
self-developed measurement *	/	18	8.654
CARE	Child Adult Relationship Experimental Index; Crittenden, as cited by Bind et al. [36]	12	5.769
CIB	Coding Interactive Behavior; Feldman [37]	12	5.769
PCERA	Parent–Child Early Relational Assessment; Clark, as cited by Anke et al. [38]	11	5.288
GRS	Global Rating Scale; Murray et al. [39]	10	4.808
IRS	Interaction Rating Scale; Field [40]	8	3.846
EAS	Emotional Availability Scales; Biringen et al., as cited by Cornish et al. [41]	7	3.365
AFFEX	Automatic Affect Expression System; Izard and Dougherty, as cited by Weinberg et al. [42]	5	2.404
Feeding Scale/SVIA	Feeding Scale; Chatoor et al. [43] (incl. Italian translation, called Scala di Valutazione Interazioni Alimentari/SVIA)	5	2.404
NICHD	Scales from the NICHD study; Owen [31]	5	2.404
MM	Mind–Mindedness; Meins and Fernyhough [44]	4	1.923
MSS	Maternal Sensitivity Scale; Ainsworth [30]	4	1.923
NCAST (NCAF and NCATS)	Nursing Child Assessment Satellite Training; Barnard; as cited by Panzarine et al. [45] Nursing Child Assessment Feeding Scale; Sumner and Spietz, as cited by Minnes et al. [46] Nursing Child Assessment Teaching Scale; Sumner and Spietz, as cited by Page [47]	4	1.923
Appearance Change Codes	Izard, as cited by Reissland and Shepherd [48]	2	0.962
Bakeman and Adamson	[49,50]	2	0.962
BMIS	Bethlem Mother–Infant Interaction Scale; Kumar and Hipwell [51]	2	0.962
Bur	Bur et al., as cited by Righetti-Veltema et al. [52]	2	0.962
GLOS	Greenspan Lieberman Observations System, Greenspan and Lieberman [53]	2	0.962
HOME	Home Observation for Measurement of the Environment; Caldwell and Bradley [54]	2	0.962
ICEP	Infant and Caregiver Engagement Manual; Weinberg and Tronick, as cited by Kaitz [55]	2	0.962
IFIRS	Iowa Family Interaction Rating Scales; Melby et al., as cited by Jaser et al. [56]	2	0.962
MBS	Mannheimer Beurteilungsskala zur Erfassung der Mutter-Kind-Interaktion; Jörg et al., as cited by Hohm et al. [57]	2	0.962
Monadic Phases	Monadic Phases; Tronick et al. [58]	2	0.962
POSER	Play Observation Scheme and Emotion Rating; Wolke, as cited by Hipwell et al. [59]	2	0.962
Siqueland et al.	[73]	2	0.962
Adaptation of HOME	as cited by [74]	1	0.481
Adaptation of MSS, Maternal Affective Expression, Baby’s Predominant Mood, and Responsiveness	referenced by Cohn et al. [75]	1	0.481
Affective Style Coding System	Doane et al., as cited by Hamilton et al. [76]	1	0.481
Ainsworth et al.	[66]	1	0.481
AIS	Affective Involvement State Scale; Cohn et al. [75]	1	0.481
AMBIANCE	Atypical Maternal Behavior Instrument for Assessment and Classification; Lyons-Ruth et al., as cited by Hobson et al. [77]	1	0.481
AMCIES	Assessment of Mother–Child-Interaction with the Etch-a-Sketch; Wolke et al., as cited by Schneider et al. [78]	1	0.481
Autonomy and Relatedness Coding System	Allen et al., as cited by Frankel-Waldheter et al. [79]	1	0.481
Based on Monadic Phases, AFFEX	referenced by Campbell et al. [80]	1	0.481
Based on MSS and NICHD	referenced by Heinisch et al. [81]	1	0.481
BATMAN	Bob and Tom’s Method of Assessing Nutrition Coding Scheme; Klesges et al. [82]	1	0.481
Baumrind	[83]	1	0.481
Behavior State System	Cohn et al., as cited by Chabrol et al. [84]	1	0.481
Bornstein	as cited by Esposito et al. [85]	1	0.481
Bornstein et al.	[86]	1	0.481
BXC	Behavioral Interaction Coder Software; Cullum et al. [87]	1	0.481
CITS	Caregiver–Infant Touch Scale; Stack, as cited by Mantis et al. [88]	1	0.481
Clarke-Stewart Rating Scale	Clarke-Stewart, as cited by Hwa-Froelich et al. [89]	1	0.481
Communication Deviance Coding Scheme	Velligan, as cited by Hamilton et al. [76]	1	0.481
Cox et al.	as cited by Craig et al. [90]	1	0.481
Datamyte	no citation found, referenced by Lovejoy [74]	1	0.481
Davies	as cited by Hart et al. [91]	1	0.481
DPICS	Dyadic Parent-Child Interaction Coding System; Eyberg [28]	1	0.481
Egeland and Hiester	as cited by Engle [92]	1	0.481
Field et al.	[93]	1	0.481
FOS	Family Observation Schedule; Sanders and Dadds [94]	1	0.481
Gaertner et al.	[95]	1	0.481
GDRS	Global Dimension Rating Scale; Hoffman, as cited by Hoffman and Drotar [96]	1	0.481
Grolnick et al.	[97]	1	0.481
Gunning et al.	[98]	1	0.481
Guthertz and Field	[99]	1	0.481
Hammen	as cited by Whaley et al. [100]	1	0.481
IDCS	Interactional Dimensions Coding System; Furman and Shomaker [101]	1	0.481
IRSS	Infant Regulatory Scoring System; Tronick and Weinberg, as cited by Beebe et al. [102]	1	0.481
Kelley and Jennings	[103]	1	0.481
Kelley et al.	[104]	1	0.481
Laing et al.	[105]	1	0.481
Landry et al.	[106]	1	0.481
Lefkowitz et al.	[107]	1	0.481
LIFE	Living in Family Environments Coding System; Hops et al., as cited by Barendse et al. [108]	1	0.481
Maternal Interaction Styles Protocol	no citation found, referenced by Defelipe et al. [109]	1	0.481
Maternal Touch	Beebe et al. [110]	1	0.481
Maternal Verbal and Nonverbal Behaviors Protocol	Defelipe et al. [109]	1	0.481
MIPCS	MACYs Infant-Parent Coding System; Earls et al., as cited by Muzik et al. [111]	1	0.481
Mocap Toolbox	Motion Capture Toolbox; Burger and Toiviainen [112]	1	0.481
Mother’s Project Rating Scale of Mother-Child Interactions	Clark et al., as cited by Goodman and Brumley [113]	1	0.481
Murray et al.	[114]	1	0.481
NICHD modified by Cox and Crnic	as cited by Azak and Raeder [115]	1	0.481
Nolen-Hoeksema et al.	[116]	1	0.481
OMCI	Observation of Mother-Child Interaction; Rasheed and Yousafzai [117]	1	0.481
Papaeliou and Trevarthen	[118]	1	0.481
Papaeliou et al.	[119]	1	0.481
PCOG	Parent–Child Observation Guide, Bernstein et al., as cited by Hans et al. [120]	1	0.481
PEM	Parental Embodied Mentalizing Coding System; Shai and Belsky [121]	1	0.481
Penman et al.	[122]	1	0.481
Pianta	as cited by Engle [92]	1	0.481
PIR-GAS	Parent–Infant Relationship Global Assessment; no citation found, referenced by Öztop and Uslu [123]	1	0.481
Procoder	Tapp [124]	1	0.481
Puckering et al.	[125]	1	0.481
Puura	no citation found; referenced by Lachmann et al. [126]	1	0.481
Rocissano et al.	[127]	1	0.481
RSIS	Rating Scale of Interactional Style; Clark and Seifer [128]	1	0.481
SACS	Simple Affect Coding System; Jabson et al., as cited by Van der Giessen and Bögels [129]	1	0.481
SCIFF	System for Coding Interactions and Family Functioning; Lindahl and Malik, as cited by Fielding [130]	1	0.481
Skuse et al.	as cited by Stein et al. [131]	1	0.481
SPAFF	Specific Affect Coding System; Gottman et al., as cited by Lee et al. [132]	1	0.481
Stack and Arnold	[133]	1	0.481
Stack and Muir	[134]	1	0.481
Stanley et al.	[135]	1	0.481
Synchrony Coding Scheme	Feldman, as cited by Granat et al. [136]	1	0.481
Tangram Coding Manual	Hudson and Rapee [137]	1	0.481
Teti and Gelfand	[138]	1	0.481
Touch Scoring Instrument	Polan and Ward [139]	1	0.481
Utterance Coding Scheme	Murray and Trevarthen; [140]	1	0.481
VITC	Vertical Interval Time Code System; Self [141]	1	0.481
Walker	as cited by Hart et al. [91]	1	0.481
Whaley et al.	[100]	1	0.481
Wolke et al.	[142]	1	0.481
Zoll et al.	as cited by Fox and Gelfand [143]	1	0.481

* Each self-developed instrument was labeled as such, without making any further distinction between different newly developed instruments. Hence, 18 researchers in total each developed a new BOI for coding.
