# Supplementary material for: Coding Dyadic Behavior in Caregiver–Child Interaction from a Clinical Psychology Perspective: How Should Multiple Instruments and Outcomes Be Dealt with?
Source: Children (Basel). 2023 Oct 31;10(11):1765. doi: 10.3390/children10111765 (PMC10670483; doi:10.3390/children10111765)
Supplement: Supplementary file 1 [file children-10-01765-s001.zip › children-2665439-supplementary.pdf]

Coding Dyadic Behavior in Caregiver–Child Interaction from a Clinical Psychology Perspective: How Should Multiple Instruments and Outcomes Be Dealt with? – Supplementary Appendix

Supplementary Table S1: Search strategy for the identification of relevant studies.

Data base	Search syntax
MEDLINE	((mother$ or maternal).ti,ab,kw. adj15 (mental disorders/ or (disorder$ or "mental illness" or "psychiatric diagnos$" or psychopathology or schizophreni$ depressi$ or dysthym$ or mania or phobi$ or agoraphobi$ or "anorexia nervosa" or "bulimia nervosa").ti,ab,kw.)) and ((mother child relations/ or maternal behavior/ or parenting/ or (parenting or caregiving or "mother-child interaction" or "mother-infant interaction" or "mother-toddler interaction" or sensitiv$ or "emotional availability" or "parent$ warmth" or "limit?setting" or hostil$ or intrusive$ or "harsh parenting" or coerci$ or overinvolve$ or "over-reactive").ti,ab,kw.) adj15 (behavioral observation techniques/ or ("behavio?ral observation" or interact$ or "free play" or "behavio?ral assessment" or "Emotional Availability Scales" or "Coding Interactive Behavior" or CIB or "CARE Index" or "Parent Child Early Relational Assessment").ti,ab,kw.))
PsycINFO	((mother$ or maternal).ti,ab,id. adj15 (mental disorders/ or (disorder$ or "mental illness" or "psychiatric diagnos$" or psychopathology or schizophreni$ or depressi$ or dysthym$ or mania or phobi$ or agoraphobi$ or "anorexia nervosa" or "bulimia nervosa").ti,ab,id.)) and ((mother child relations/ or parenting/ or (parenting or caregiving or "mother-child interaction" or "mother-infant interaction" or "mother-toddler interaction" or sensitiv$ or "emotional availability" or "parent$ warmth" or "limit?setting" or hostil$ or intrusive$ or "harsh parenting" or coerci$ or overinvolve$ or "over-reactive").ti,ab,id.) adj15 (observation methods/ or behavioral assessment/ or ("behavio?ral observation" or interact$ or "free?play" or "behavio?ral assessment" or "Emotional Availability Scales" or "Coding Interactive Behavior" or CIB or "CARE Index" or "Parent Child Early Relational Assessment").ti,ab,id.))
PubMed	(mother*[Title/Abstract] OR maternal[Title/Abstract]) AND ("Mental Health"[MeSH Terms] OR "Mental Disorders"[MeSH Terms] OR "Diagnosis/psychology"[MeSH Terms] OR disorder*[Title/Abstract] OR “mental illness”[Title/Abstract] OR “psychiatric diagnos*”[Title/Abstract] OR psychopathology[Title/Abstract] OR schizophreni*[Title/Abstract] OR depressi*[Title/Abstract] OR dysthym*[Title/Abstract] OR mania[Title/Abstract] OR phobi*[Title/Abstract] OR agoraphobi*[Title/Abstract] OR “anorexia nervosa”[Title/Abstract] OR “bulimia nervosa”[Title/Abstract]) AND ("Maternal Behavior"[MeSH Terms] OR "Child Rearing"[MeSH Terms] OR "Parenting"[MeSH Terms] OR "Mother-Child Relations"[MeSH Terms] OR parenting[Title/Abstract] OR caregiving[Title/Abstract] OR “mother-child interaction”[Title/Abstract] OR “mother-infant interaction”[Title/Abstract] OR “mother-toddler interaction[“Title/Abstract] OR sensitiv*[Title/Abstract] OR “emotional availability”[Title/Abstract] OR “parent* warmth”[Title/Abstract] OR “limit setting”[Title/Abstract] OR hostil*[Title/Abstract] OR intrusive*[Title/Abstract] OR “harsh parenting”[Title/Abstract] OR coerci*[Title/Abstract] OR overinvolve*[Title/Abstract] OR “over-reactive”[Title/Abstract) AND ("Behavior Observation Techniques"[MeSH Terms] OR “behavioral observation”[Title/Abstract] OR interact*[Title/Abstract] OR “free play”[Title/Abstract] OR “behavioral assessment”[Title/Abstract] OR

	“Emotional Availability Scales”[Title/Abstract] OR “Coding Interactive Behavior”[Title/Abstract] OR CIB OR “CARE Index”[Title/Abstract] OR “Parent Child Early Relational Assessment”[Title/Abstract])
Scopus	TITLE-ABS-KEY (((((disorder* W/15 mother*) OR ("mental illness" W/15 mother*) OR ("psychiatric diagnos*" W/15 mother*) OR (psychopathology W/15 mother*) OR (schizophreni* W/15 mother*) OR (depressi* W/15 mother*) OR (dysthym* W/15 mother*) OR (mania W/15 mother*) OR (phobi* W/15 mother*) OR (agoraphobi* W/15 mother*) OR ("anorexia nervosa" W/15 mother*) OR ("bulimia nervosa" W/15 mother*)) OR ((disorder* W/15 maternal) OR ("mental illness" W/15 maternal) OR ("psychiatric diagnos*" W/15 maternal) OR (psychopathology W/15 maternal) OR (schizophreni* W/15 maternal) OR (depressi* W/15 maternal) OR (dysthym* W/15 maternal) OR (mania W/15 maternal) OR (phobi* W/15 maternal) OR (agoraphobi* W/15 maternal) OR ("anorexia nervosa" W/15 maternal) OR ("bulimia nervosa" W/15 maternal))) AND (parenting OR caregiving OR "mother-child interaction" OR "mother-infant interaction" OR "mother-toddler interaction" OR sensitiv* OR "emotional availability" OR "parent* warmth" OR "limit setting" OR hostil* OR intrusive* OR "harsh parenting" OR coerci* OR overinvolve* OR "over-reactive") AND ("behavioral observation" OR interact* OR "free play" OR "behavioral assessment" OR "Emotional Availability Scales" OR "Coding Interactive Behavior" OR "CIB" OR "CARE Index" OR "Parent Child Early Relational Assessment"))
Web of Science	*[to search in title, abstract, author keywords and Keywords Plus, “Topic” was preselected in the search field of Web of Science]* ((mother* OR maternal) NEAR/15 (disorder* OR "mental illness" OR "psychiatric diagnos*" OR psychopathology OR schizophreni* OR depressi* OR dysthym* OR mania OR phobi* OR agoraphobi* OR "anorexia nervosa" OR "bulimia nervosa")) AND ((parenting OR caregiving OR "mother-child interaction" OR "mother-infant interaction" OR "mother-toddler interaction" OR sensitiv* OR "emotional availability" OR "parent* warmth" OR "limit setting" OR hostil* OR intrusive* OR "harsh parenting" OR coerci* OR overinvolve* OR "over-reactive") NEAR/15 ("behavio$ral observation" OR interact* OR "free play" OR "behavio$ral assessment" OR "Emotional Availability Scales" OR "Coding Interactive Behavior" OR CIB OR "CARE Index" OR "Parent Child Early Relational Assessment"))

Supplementary Table S2: Stimulus coordinates of the SST terms.

Term	Dimension 1	Dimension 2	Instrument*
IndirectCommand	-1.4899	-0.1266	DPICS
Sensitivity_2	1.4285	0.3705	MSS
SensitivityDistress	1.4236	0.3621	NICHD
DescriptiveQuestion	-1.4173	0.6791	DPICS
SensitivityNondistress	1.4093	0.2589	NICHD
DirectCommand	-1.409	-0.3505	DPICS
Questions	-1.4081	0.6909	DPICS
Sensitivity_4	1.3955	0.3693	EAS
InformationQuestion	-1.3887	0.6643	DPICS
Availability	1.3843	-0.0827	MSS
NeutralTalk	-1.3551	0.9151	DPICS

Command	-1.353	-0.3293	DPICS
Sensitivity_3	1.3465	0.4356	CIB
Reflection	-1.3065	0.7449	DPICS
Flatness	1.2947	-0.0899	NICHD
Structuring	-1.291	-0.6502	EAS
AcceptanceRejection	1.2879	0.5634	MSS
LabeledPraise	-1.2824	0.7012	DPICS
Sensitivity_1	1.2621	0.5839	CARE
BehaviorDescription	-1.2119	0.9304	DPICS
NegativeTalk	-1.2066	-0.4957	DPICS
PositiveTouch	1.0834	0.7471	DPICS
PositiveRegard	1.08	0.0616	NICHD
Unresponsiveness	1.0254	0.0228	CARE
Praise	-1.0155	0.8158	DPICS
Detachment	0.9159	-0.7882	NICHD
LimitSetting**	-1.0976	-1.0951	CIB
Touch	-0.023	1.7526	DPICS
Intrusiveness_1	0.3861	-1.3663	NICHD
Intrusiveness_2	0.3408	-1.3568	CIB
Nonintrusiveness	0.3414	-1.3363	EAS
Cooperation	0.3495	-1.2869	MSS
Nonhostility	0.952	-1.1427	EAS
Control	-0.9675	-1.1208	CARE
Stimulation	-0.2584	1.0682	NICHD
NegativeTouch	0.8874	-1.0402	DPICS
NegativeRegard	0.8087	-1.0395	NICHD
UnlabeledPraise	-0.9216	0.96	DPICS

* CARE: Child Adult Relationship Experimental Index, CIB: Coding Interactive Behavior, DPICS: Dyadic Parent–Child Interaction Coding System, EAS: Emotional Availability Scales, MSS: Maternal Sensitivity Scale, NICHD: scales from the NICHD study.

** Because *LimitSetting* loaded up on both dimensions, we refrained from assigning it to one dimension and considered the term when interpreting the content of both dimensions.

Supplementary Figure S1: SST solution with three dimensions

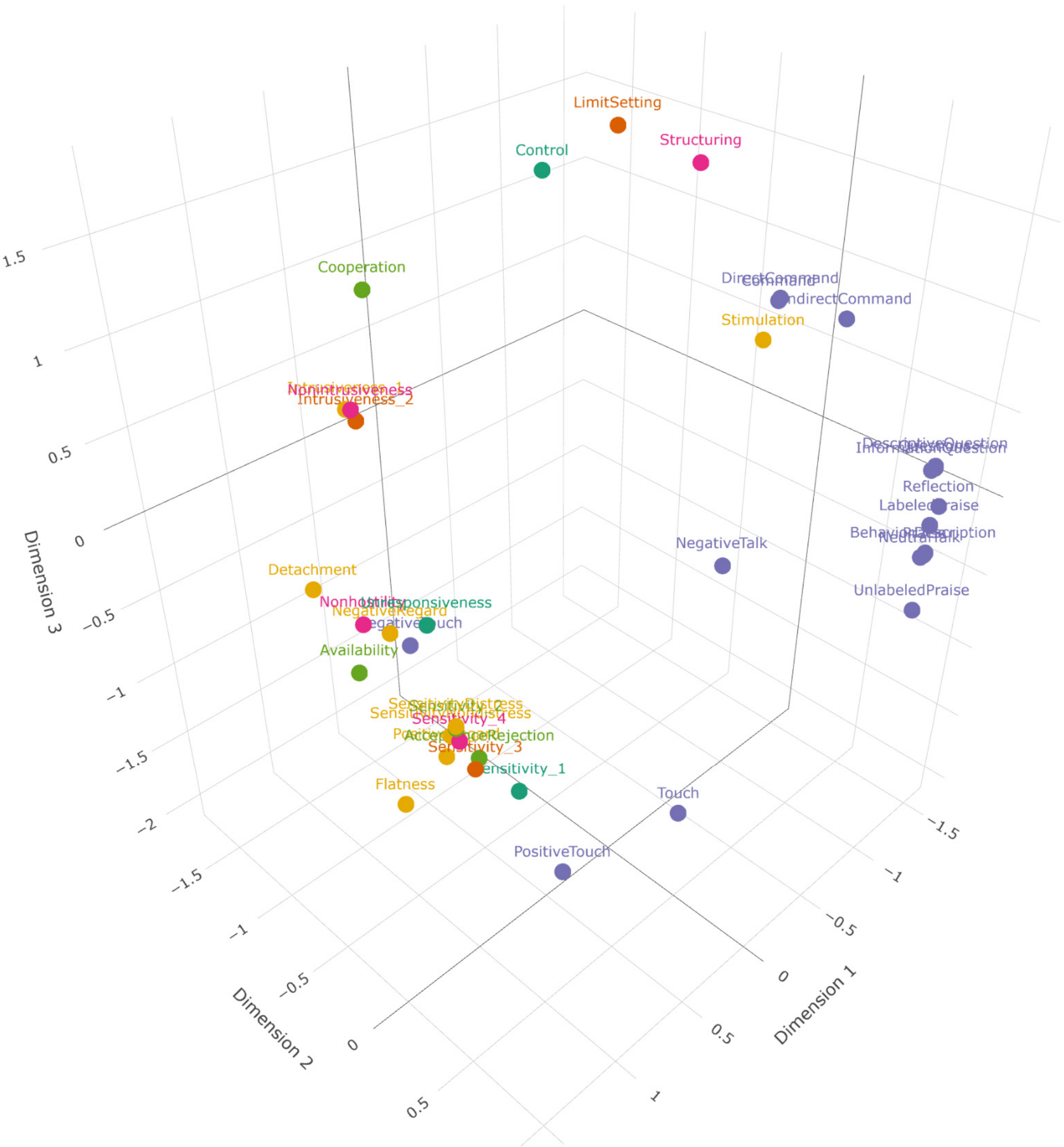

Figure S1. SST solution with three dimensions.
Note. An interactive version of this three-dimensional solution is available in the repository (see below).

The interactive figure of a three-dimensional SST solution is available in the repository: https://osf.io/j2x7r/?view_only=178f098222934be98a5471683ca3426d
For access, the HTML code must be downloaded and opened from the download area, which automatically redirects to the three-dimensional solution.
